# Draft Genome Sequences of Three Rhizospheric Plant Growth-Promoting Bacteria

**DOI:** 10.1128/MRA.00455-19

**Published:** 2019-06-27

**Authors:** Olubukola Oluranti Babalola, Ayansina Segun Ayangbenro, Oluwaseyi Samuel Olanrewaju

**Affiliations:** a Food Security and Safety Niche Area, Faculty of Natural Science and Agriculture, North-West University, Potchefstroom, South Africa; University of Rochester School of Medicine and Dentistry

## Abstract

Here, we report the draft genome sequences of Bacillus subtilis A1, Sphingobacterium sp. strain A3, and Pseudomonas sp. strain A29; Sphingobacterium sp. A3 and Pseudomonas sp. A29 were identified as Bacillus velezensis strain A3 and Bacillus subtilis strain A29, respectively, after a quality control check of the whole-genome sequences deposited in the NCBI database. These bacteria exhibit tremendous production of siderophores and significant antimicrobial potential. When inoculated on maize, these isolates increase its yield.

## ANNOUNCEMENT

Plant growth promoting-rhizobacteria (PGPR) have become so important to agricultural sustainability and food security. The increasing human population and hazardous impact of chemical fertilizers on the environment have favored the use of PGPR as biofertilizer and biocontrol agents.

Bacillus subtilis A1, Bacillus velezensis strain A3, and Bacillus subtilis strain A29, reported here, were isolated from maize rhizosphere from a maize field (25°49′S, 27°5′E) in Mafikeng, South Africa. For the isolation of rhizobacteria, 10 g of rhizospheric soil was suspended in 90 ml sterile distilled water. The serial dilution method was used for further analysis of the prepared soil suspension. Suitable dilutions (10^−2^, 10^−4^, and 10^−6^) were plated in triplicate onto Luria-Bertani (LB) agar (Sigma-Aldrich) to isolate rhizobacteria using standard microbiological isolation techniques, and plates were incubated at 25°C for 24 h. Bacterial colonies were subcultured and purified by streaking onto fresh LB agar plates. These isolates were first identified by 16S rRNA sequencing.

The genomic DNA was extracted from overnight cultures in LB medium (1) using a ZR soil microbe DNA MiniPrep extraction kit (Zymo Research, USA), following the manufacturer’s instructions. The DNA quality and quantity were determined using a NanoDrop Lite spectrophotometer (Thermo Fisher Scientific, CA, USA). The genomes of the strains were sequenced on an Illumina HiSeq sequencer at Molecular Research (MR DNA), Shallowater, TX. The libraries were prepared using Kapa HyperPlus kits (Roche), following the manufacturer’s user guide. The initial concentration of DNA was evaluated using the Qubit double-stranded DNA (dsDNA) high-sensitivity (HS) assay kit (Life Technologies). A total of 25 ng of DNA was used to prepare the libraries. The protocol starts with enzymatic fragmentation to produce dsDNA fragments, followed by end repair and A-tailing to produce end-repaired 5′-phosphorylated 3′-deoxyribosyladenine (dA)-tailed dsDNA fragments. In the adapter ligation step, dsDNA adapters are ligated to 3′-dA-tailed molecules. The final step is library amplification, which employs high-fidelity, low-bias PCR to amplify library fragments carrying appropriate adapter sequences on both ends. Following the library preparation, the final concentrations of the libraries were measured using the Qubit dsDNA HS assay kit (Life Technologies), and the average library size was determined using the Agilent 2100 Bioanalyzer (Agilent Technologies). Bacillus subtilis A1, Bacillus velezensis strain A3, and Bacillus subtilis strain A29 DNA concentrations are 114.0, 84.8, and 187.0 ng/μl, respectively, while the final library DNA concentrations are 62.0, 62.0, and 58.8 ng/μl, respectively. The average library sizes of Bacillus subtilis A1, Bacillus velezensis strain A3, and Bacillus subtilis strain A29 are 680, 694, and 695 bp, respectively. The libraries were pooled, diluted (to 9.0 pM), and paired-end sequenced for 500 cycles using the HiSeq system (Illumina), with an average read length of 2 × 250 bp.

The raw sequences were processed to obtain high-quality reads using the KBase (2) platform. The quality of the reads was checked using FastQC (v.1.0.4) (3). The reads were trimmed to remove adapters and low-quality sequences using Trimmomatic (v.0.36) (4), with the default parameters. The reads were assembled by *de novo* assembly using SPAdes v.3.12.0 (5), with the default parameters. The draft genomes of Bacillus subtilis A1, Bacillus velezensis strain A3, and Bacillus subtilis strain A29 include 23, 451, and 22 contigs, respectively, with *N*_50_ contig sizes of 2,070,070 bp, 980,387 bp, and 1,061,166 bp, respectively. The genome sizes are 4,065,600 bp, 4,154,675 bp, and 4,065,078 bp, respectively; the G+C content for both Bacillus subtilis A1 and Bacillus subtilis strain A29 is 43.8%, and that of Bacillus velezensis strain A3 is 46.4%. Gene function prediction was performed using the Rapid Annotations using Subsystems Technology (RAST v.2.0) server (http://rast.nmpdr.org) (6), followed by annotation using the NCBI Prokaryotic Genome Annotation Pipeline (PGAP v.4.7) (7). The results are summarized in Table 1.

**TABLE 1:** Annotation results of the strains

Parameter	Value for isolate:		
	Bacillus subtilis A1	Bacillus velezensis strain A3	Bacillus subtilis strain A29
No. of coding genes	4,060	4,258	4,063
No. of total RNAs	105	101	105
No. of rRNAs	19	16	19
No. of tRNAs	81	80	81
No. of pseudogenes	103	118	100

*In silico* analysis using antiSMASH v.3.0 (8) and RAST revealed the presence of siderophore gene clusters and genes involved with the production of indole acetic acid (IAA), phosphate, and nitrogen metabolism, which are all associated with plant growth promotion and biocontrol (9–12) in the three isolates. Clusters implicated in the biosynthesis of antifungal and antimicrobial compounds, such as bacillaene (100%), fengycin (100%), bacillibactin (100%), subtilosin A (100%), bacilysin (100%), surfactin (43%), difficidin (60%), and macrolactin (60%), were all present. The complete genomes of these strains will be helpful to further study the mechanisms of plant growth promotion and biocontrol at the molecular level for further biotechnological application.

### Data availability.

These whole-genome shotgun projects have been deposited at DDBJ/ENA/GenBank under the accession numbers SHOB00000000, SHOC00000000, and SHOD00000000 for Bacillus subtilis A1, Bacillus velezensis A3, and Bacillus subtilis A29, respectively. The versions described in the manuscript are the first versions, SHOB010000001, SHOC010000001, and SHOD01000000, respectively. The BioProject accession numbers are PRJNA516328, PRJNA516332, and PRJNA516331, respectively. The Sequence Read Archive (SRA) has accession numbers SRR8540661, SRR8550002, and SRR8541016. SRR8550002 and SRR8541016 are links to the initial identified isolates, which are *Sphingobacterium* sp. strain A3 and *Pseudomonas* sp. strain A29, respectively.
